# Combinational Deletions of *MGF110-9L* and *MGF505-7R* Genes from the African Swine Fever Virus Inhibit TBK1 Degradation by an Autophagy Activator PIK3C2B To Promote Type I Interferon Production

**DOI:** 10.1128/jvi.00228-23

**Published:** 2023-05-10

**Authors:** Guoqiang Zhu, Jingjing Ren, Dan Li, Yi Ru, Xiaodong Qin, Tao Feng, Hong Tian, Bingzhou Lu, Dongfang Shi, Zhengwang Shi, Wenping Yang, Haixue Zheng

**Affiliations:** a State Key Laboratory for Animal Disease Control and Prevention, College of Veterinary Medicine, Lanzhou University, Lanzhou Veterinary Research Institute, Chinese Academy of Agricultural Sciences, Lanzhou, China; b College of Veterinary Medicine, Northeast Agricultural University, Harbin, China; Lerner Research Institute, Cleveland Clinic

**Keywords:** African swine fever virus, RNA-seq, TBK1, PIK3C2B, autophagic degradation

## Abstract

African swine fever (ASF), caused by the African swine fever virus (ASFV), is a transboundary infectious disease of domestic pigs and wild boars, resulting in significant swine production losses. Currently, no effective commercial ASF vaccines or therapeutic options are available. A previous study has shown that deletions of ASFV *MGF110-9L* and *MGF505-7R* genes (ASFV-Δ110-9L/505-7R) attenuated virulence in pigs and provided complete protection against parental lethal ASFV CN/GS/2018 (wild-type ASFV [ASFV-WT]) challenge, but the underlying mechanism is unclear. This study found that ASFV-Δ110-9L/505-7R weakened TBK1 degradation compared with ASFV-WT through RNA sequencing (RNA-seq) and Western blotting analyses. Furthermore, we confirmed that ASFV-Δ110-9L/505-7R blocked the degradation of TBK1 through the autophagy pathway. We also identified that the downregulation of an autophagy-related protein PIK3C2B was involved in the inhibition of TBK1 degradation induced by ASFV-Δ110-9L/505-7R. Additionally, we also confirmed that PIK3C2B promoted ASFV-Δ110-9L/505-7R replication *in vitro*. Together, this study elucidated a novel mechanism of virulence change of ASFV-Δ110-9L/505-7R, revealing a new mechanism of ASF live attenuated vaccines (LAVs) and providing theoretical guidance for the development of ASF vaccines.

**IMPORTANCE** African swine fever (ASF) is a contagious and lethal hemorrhagic disease of pigs caused by the African swine fever virus (ASFV), leading to significant economic consequences for the global pig industry. The development of an effective and safe ASF vaccine has been unsuccessful. Previous studies have shown that live attenuated vaccines (LAVs) of ASFV are the most effective vaccine candidates to prevent ASF. Understanding the host responses caused by LAVs of ASFV is important in optimizing vaccine design and diversifying the resources available to control ASF. Recently, our laboratory found that the live attenuated ASFV-Δ110-9L/505-7R provided complete protection against parental ASFV-WT challenge. This study further demonstrated that ASFV-Δ110-9L/505-7R inhibits TBK1 degradation mediated by an autophagy activator PIK3C2B to increase type I interferon production. These results revealed an important mechanism for candidate vaccine ASFV-Δ110-9L/505-7R, providing strategies for exploring the virulence of multigene-deleted live attenuated ASFV strains and the development of vaccines.

## INTRODUCTION

African swine fever (ASF) is an acute, febrile, hemorrhagic, highly contacting, and pathogenic transboundary disease caused by the African swine fever virus (ASFV). However, no safe and effective commercial vaccines and drugs are available for controlling ASF (1–3). ASFV is an enveloped virus containing a double-stranded DNA genome of approximately 180 to 190 kbp, encoding more than 150 viral proteins. ASFV particles are enveloped by a multilayer structure consisting of a nucleoid, core shell, inner lipid envelope, icosahedral protein capsid, and external lipid envelope (4). In addition, the proteomic analysis showed that mature ASFV particles contain a large number of viral (~70) and host (>20) proteins simultaneously (5). The complex structures of ASFV particles suggest that ASFV has complicated replication and host-virus interaction mechanisms.

ASFV has evolved counteracting strategies to evade host immune response and to maintain efficient replication (6, 7). A series of ASFV genes have been reported to play a crucial role in the invasion and regulation of host immune responses (8, 9). Of those regulated genes, multigene family (MGF) members, including MGF100, MGF110, MGF300, MGF360, and MGF505, are the main genes that regulate host immune responses, host range, antigenic variation, and viral virulence (9–12). For example, MGF360-9L involves the effects of ASFV pathogenicity by the targeted degradation of STAT1 and STAT2 (13). MGF360-11L antagonizes the host innate antiviral immune response by degrading TBK1 and IRF7 and inhibiting interleukin 1 beta (IL-1β) production (14). MGF360-12L is critical for maintaining efficient replication of ASFV in macrophages (12). Additionally, MGF505-7R and MGF110-9L are important well-characterized interferon inhibitors (15). MGF505-7R inhibits type I interferon production by interacting with multiple immune pathway key nodule molecules such as IRF3, IRF7, TBK1, NF-κB, and STING (16–18). MGF505-7R is also involved in regulating host inflammatory response (19). MGF110-9L is critical for virus replication and a virulence factor (15, 20).

Some other ASFV genes also play pivotal roles in regulating host antiviral response and viral replication and are considered effective live attenuated vaccine (LAV) candidate targets, such as *I177L* (21), *B119L* (22), *DP96R* (23), *EP402R* (24), *I226R* (25), *A137R* (26), and *E184L* (27). Of these, ASFV-G-ΔI177L offers 100% protection of pigs against the epidemiologically lethal ASFV Georgia isolate challenge (21, 28), but the biosecurity risks of ASFV-G-ΔI177L in domestic pigs, especially in intensive pig farms, cannot be completely excluded (29).

According to the experience and understanding of previous studies, gene-deleted live attenuated vaccines of ASFV are still the most effective vaccine candidates to prevent ASF. However, stacks of research papers have suggested that deletion of a single gene is insufficient to attenuate the virulence of virus and against challenge of parental ASFV (29). Recently, our laboratory has reported that the live attenuated ASFV-Δ110-9L/505-7R provided complete protection against parental ASFV challenge. However, the attenuated mechanism of ASFV-Δ110-9L/505-7R is currently unclear (15). In the present study, we found that the difference in virulence between wild-type ASFV (ASFV-WT) and ASFV-Δ110-9L/505-7R strains is mainly caused by the difference in innate immune responses. Further studies revealed that ASFV-WT and ASFV-Δ110-9L/505-7R infection led to the differential expression of TBK1 by recruiting an autophagy activator, PIK3C2B. This study elucidated a novel mechanism of virulence change of ASFV-Δ110-9L/505-7R, providing a theoretical basis for studying the virulence of multigene-deleted live attenuated ASFV strains and the development of vaccines.

## RESULTS

### Experimental design and RNA-seq data quality check.

**FIG 1:**
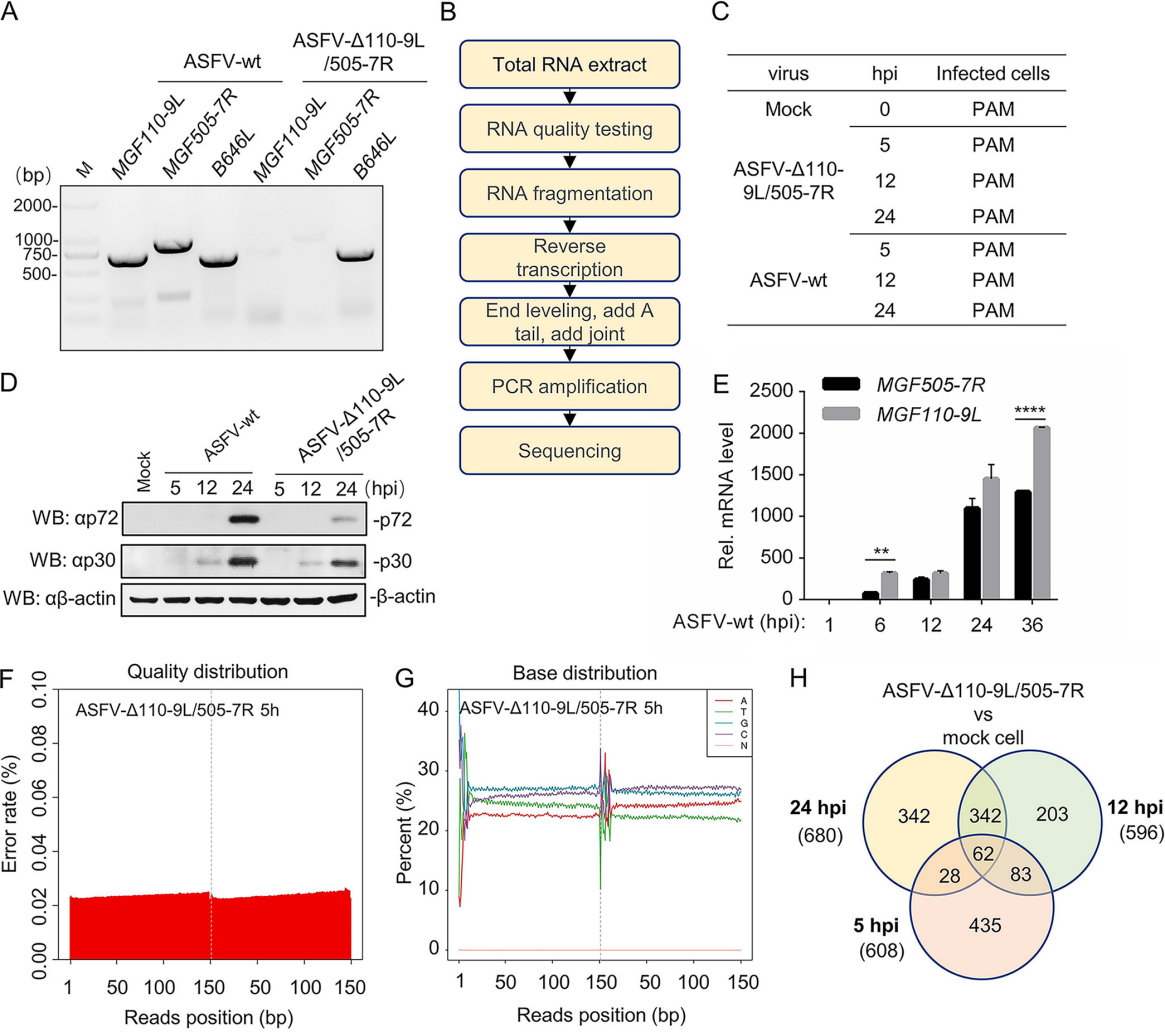
Experimental design and quality testing of RNA-seq. (A) PCR analysis of ASFV-Δ110-9L/5T05-7R purity utilizing primers MGF110-9L-F/R and MGF360-9L-F/R. Meanwhile, the *B646L* gene was set as a positive control. (B and C) Strategy of RNA-seq and experimental design. (B) Cells were collected for RNA sequencing analysis according to the standard procedures. (C) PAM cells were infected with ASFV-WT and ASFV-Δ110-9L/505-7R at an MOI of 1 for 5, 12, and 24 h. (D) Evaluation of ASFV viral proteins p72 and p30 in ASFV-WT- and ASFV-Δ110-9L/505-7R-infected PAM cells. PAM cells were infected with ASFV-WT and ASFV-Δ110-9L/505-7R at an MOI of 1 for 5, 12, and 24 h. Cells were collected, and expressions of p72 and p30 were evaluated by Western blotting using antibodies p72 and p30. (E) Transcription levels of *MGF110-9L* and *MGF505-7R* genes during ASFV infection. PAM cells were infected with ASFV-WT and ASFV-Δ110-9L/505-7R at an MOI of 1 for 1, 6, 12, 24, and 36 h. Then, cells were collected and the transcription levels of *MGF110-9L* and *MGF505-7R* genes were evaluated by qRT-PCR. (F and G) The quality distribution (F) and base distribution (G) of sequencing data were analyzed by Phred. (H) Venn diagram of differentially expressed genes (DEGs) between mock and ASFV-Δ110-9L/505-7R-infected cells. The pink, green, and yellow circles represent the DEGs at 5, 12, and 24 hpi, respectively. PAMs, porcine alveolar macrophages; hpi, hours postinfection; MOI, multiplicity of infection; qRT-PCR, quantitative real-time PCR. **, *P < *0.01; ****, *P < *0.001.

Our laboratory's recent study has shown that combinational deletions of *MGF110-9L* and *MGF505-7R* from ASFV-WT (ASFV-Δ110-9L/505-7R) significantly attenuated ASFV-WT virulence and provided effective protection against parental ASFV challenge (15); however, the underlying mechanism remains unclear. To elucidate the potential mechanism of ASFV-Δ110-9L/505-7R attenuated virulence, the purity of ASFV-Δ110-9L/505-7R was first detected by PCR utilizing primers MGF110-9L-F/R, MGF505-7R-F/R, and B647L-F/R (Table 1). The results showed that no bands of MGF110-9L or MGF505-7R were detected in ASFV-Δ110-9L/505-7R (Fig. 1A), suggesting the combinational deletions of the two target genes. To further understand host dynamic changes of transcriptional states in ASFV-WT- and ASFV-Δ110-9L/505-7R-infected porcine alveolar macrophages (PAMs), RNA sequencing (RNA-seq) analysis was performed (Fig. 1B). ASFV-WT- and ASFV-Δ110-9L/505-7R-infected PAM cells were collected at different time points (Fig. 1C). The expression of p72 (coded by the viral late gene *B646L*) and p30 (coded by the viral early gene *CP204L*) was detected in the ASFV-WT- and ASFV-Δ110-9L/505-7R-infected cells by Western blotting, and the results showed that the expression of these two proteins in ASFV-Δ110-9L/505-7R-infected cells was weaker than that in ASFV-WT-infected cells (Fig. 1D). In order to explore the difference in transcription level of the *MGF110-9L* or *MGF505-7R* genes during ASFV infection, quantitative real-time PCR (qRT-PCR) analysis was performed, and the results showed that the transcription level of MGF110-9L was significantly higher and earlier than that of MGF505-7R at 6 and 36 hours postinfection (hpi) (Fig. 1E). In addition, the quality distribution and base distribution of sequencing data met the experimental requirements (Fig. 1F and G), and the alignment rate of reads to the reference genome also met the requirements (Table 2). The total number of differentially expressed genes (DEGs) between ASFV-Δ110-9L/505-7R-infected cells and mock cells at 5, 12, and 24 h hpi was 608, 596, and 680, respectively (*P* < 0.05 and |log_2_FC| > 1) (Fig. 1H).

**TABLE 1:** Information on PCR primers used for testing purity

Primer name	Sequence (5′–3)′	Fragment size (bp)
MGF110-9L-F	CATACATACACCGGTCATGC	695
MGF110-9L-R	AGGAACAGGAGAGTTACCAATG	695
MGF505-7R-F	GCTTATAGAGCATGATCTTAC	885
MGF505-7R-R	AGTCTTATCACAAGAGTAGAG	885
B646L-F	AAGGGAATGGATACTGAGGG	638
B646L-R	AACGGATATGACTGGGACAA	638

**TABLE 2:** Alignment rate of reads to the reference genome

Sample name	Total no. of reads	Total no. (%) of mapped reads	No. (%) of multiple mapped reads	No. (%) of uniquely mapped reads	Read 1 (no. [%])	Read 2 (no. [%])	No. (%) of nonspliced reads	No. (%) of spliced reads	No. (%) of reads mapped in proper pairs
Mock	48,840,654	47,432,538 (97.12)	2,355,233 (4.82)	45,077,305 (92.29)	22,565,747 (46.20)	22,511,558 (46.09)	24,873,115 (50.93)	20,204,190 (41.37)	43,801,268 (89.68)
A-WT 5 h	45,561,480	44,417,863 (97.49)	1,607,918 (3.53)	42,809,945 (93.96)	21,429,880 (47.04)	21,380,065 (46.93)	23,774,749 (52.18)	19,035,196 (41.78)	41,677,138 (91.47)
A-WT 12 h	48,162,550	46,579,302 (96.71)	1,441,484 (2.99)	45,137,818 (93.72)	22,588,835 (46.90)	22,548,983 (46.82)	24,212,161 (50.27)	20,925,657 (43.45)	43,955,554 (91.27)
A-WT 24 h	48,823,380	45,698,836 (93.60)	1,877,607 (3.85)	43,821,229 (89.75)	21,932,965 (44.92)	21,888,264 (44.83)	22,814,552 (46.73)	21,006,677 (43.03)	42,628,850 (87.31)
A-Δ7R/9L 5 h	49,809,914	48,641,506 (97.65)	1,850,761 (3.72)	46,790,745 (93.94)	23,418,286 (47.02)	23,372,459 (46.92)	24,711,056 (49.61)	22,079,689 (44.33)	45,597,196 (91.54)
A-Δ7R/9L 12 h	46,744,370	45,829,901 (98.04)	1,419,285 (3.04)	44,410,616 (95.01)	22,218,836 (47.53)	22,191,780 (47.47)	23,305,459 (49.86)	21,105,157 (45.15)	43,228,122 (92.48)
A-Δ7R/9L 24 h	48,543,928	47,613,011 (98.08)	1,417,278 (2.92)	46,195,733 (95.16)	23,108,744 (47.60)	23,086,989 (47.56)	24,381,792 (50.23)	21,813,941 (44.94)	45,011,288 (92.72)

### DEGs are mainly enriched in cytokine response and interaction.

**FIG 2:**
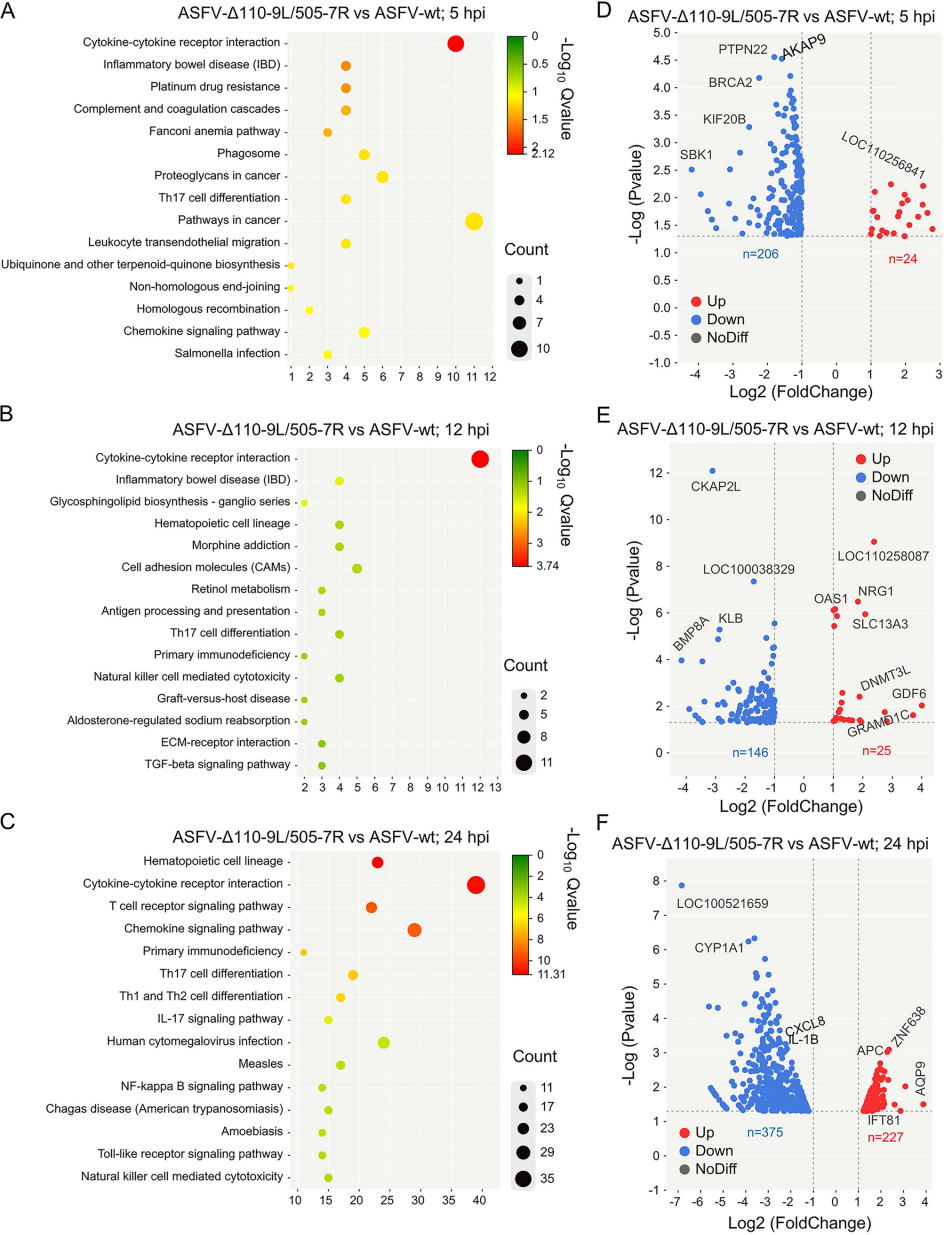
Analyses of KEGG pathway and Volcano plot for DEGs. (A to C) KEGG pathway analysis for DEGs (*P* < 0.05 and |log_2_FC| > 1) between ASFV-WT- and ASFV-Δ110-9L/505-7R-infected PAM cells at 5 (A), 12 (B), and 24 (C) hpi. The dots’ color and size represent the fold changes and the number of enriched DEGs, respectively. (D to F) Volcano plots of DEGs (*P* < 0.05 and |log_2_FC| > 1) between ASFV-WT- and ASFV-Δ110-9L/505-7R-infected PAM cells at 5 (D), 12 (E), and 24 (F) hpi. The red dots represent the upregulated DEGs, and the blue dots represent the downregulated DEGs. The gray dots indicate no significant difference. DEGs, differentially expressed genes; hpi, hours postinfection; FC, fold change; KEGG, Kyoto Encyclopedia of Genes and Genomes.

Kyoto Encyclopedia of Genes and Genomes (KEGG) pathway analysis was conducted to further explore the differential changes in biological signaling pathways during ASFV-WT and ASFV-Δ110-9L/505-7R infection, and the results showed that DEGs were mainly enriched in the cytokine-cytokine receptor interaction at different stages of infection (Fig. 2A to C). At the later stages of infection (24 hpi), DEGs were also enriched in the NF-κB signaling pathway and other immune-related pathways (Fig. 2C). Notably, ASFV-Δ110-9L/505-7R infection induced more DEGs than ASFV-WT infection, among which 206, 145, and 376 DEGs were downregulated and 24, 24, and 227 DEGs were upregulated at 5, 12, and 24 hpi, respectively (Fig. 2D to F). These results preliminarily indicated that ASFV-Δ110-9L/505-7R infection induces stronger host responses than ASFV-WT infection.

### TBK1 reduction is significantly suppressed in ASFV-Δ110-9L/505-7R-infected PAM cells.

**FIG 3:**
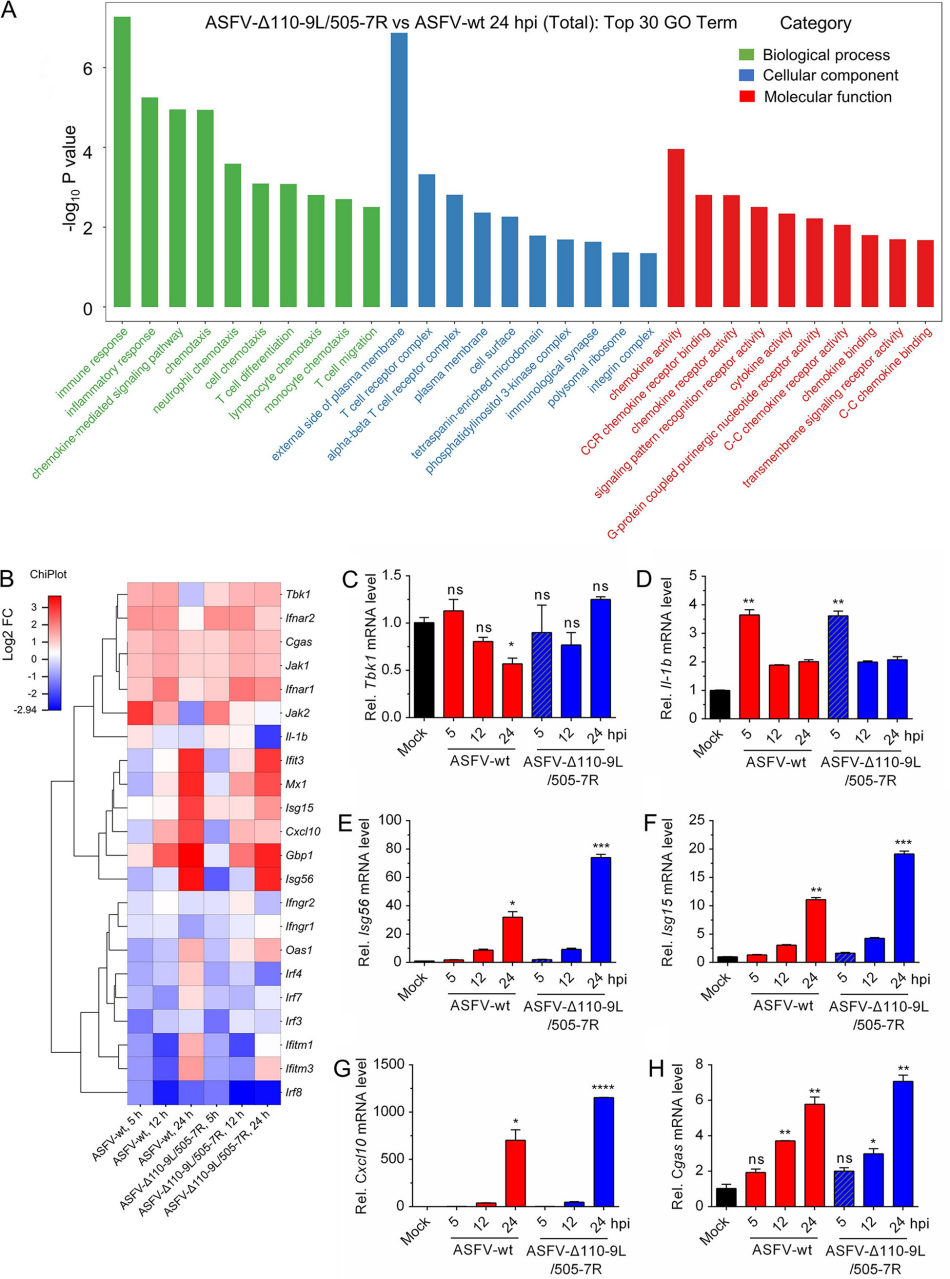
Comparison of RNA-seq results during ASFV-Δ110-9L/505-7R or ASFV-WT infection. (A) Gene ontology (GO) function enrichment analysis of DEGs between ASFV-WT- and ASFV-Δ110-9L/505-7R-infected cells at 24 hpi. (B) Heatmap of DEGs between ASFV-WT- and ASFV-Δ110-9L/505-7R-infected cells. A total of 22 DEGs involved in the IFN pathway were selected and displayed in the heatmap. The color depicts the fold change (FC) at the indicated time points. (C to H) qRT-PCR validation for several obvious DEGs presented in the heatmap. PAM cells were infected with ASFV-WT or ASFV-Δ110-9L/505-7R (MOI of 1) for 5, 12, and 24 h. Then, cells were collected, and transcription levels of *Tbk1* (C), *Il-1b* (D), *Isg56* (E), *Isg15* (F), *Cxcl10* (G), and *Cgas* (H) were assessed by qRT-PCR. DEGs, differentially expressed genes; hpi, hours postinfection; qRT-PCR, quantitative real-time PCR; MOI, multiplicity of infection. *, *P < *0.05; **, *P < *0.01; ***, *P < *0.005; ns, no significance.

To explore the function of DEGs in host response, gene ontology (GO) function analysis was performed, and the top 10 GO items are presented in Fig. 3A according to the −log_10_ *P* value. The results showed that the immune responses are the most significantly enriched terms in biological processes (Fig. 3A). Hence, 22 DEGs (*Tbk1*, etc.) associated with immune responses were selected and clustered in the heatmap (Fig. 3B). Furthermore, qRT-PCR analysis was carried out to validate the expression of 6 DEGs (*Tbk1*, *Il-1b*, *Isg56*, *Isg15*, *Cxcl10*, and *Cgas*). Compared with ASFV-WT infection, ASFV-Δ110-9L/505-7R infection significantly blocked *Tbk1* reduction at 24 hpi (Fig. 3B and C). Both ASFV-Δ110-9L/505-7R and ASFV-WT infection significantly enhanced *Il-1b* expression at 5 hpi, whereas no significant difference was observed at 12 and 24 hpi (Fig. 3D). Also, expression of *Cxcl10*, *Isg56*, and *Isg15* genes was higher in ASFV-Δ110-9L/505-7R-infected PAM cells than that in ASFV-WT-infected PAM cells (Fig. 3E to G). Both ASFV-Δ110-9L/505-7R and ASFV-WT infection significantly enhanced *Cgas* expression at 12 and 24 hpi, whereas no significant difference was observed at 5 hpi (Fig. 3H). These results indicated that ASFV-Δ110-9L/505-7R induces a stronger immune response than ASFV-WT.

### ASFV-Δ110-9L/505-7R infection enhanced type I IFN production.

Previously, it has been demonstrated that ASFV MGF505-7R inhibited the cGAS-STING signaling pathway, and the *MGF505-7R* gene-deleted ASFV strain increased interferon beta (IFN-β) production (18, 30). The *MGF110-9L*-deficient mutant strain results in attenuated virulence in pigs (20). Remarkably, ASFV-Δ110-9L/505-7R has also been reported to attenuate virulence in pigs and provided complete protection against ASFV-WT challenge, and it was highly resistant to genetic modification (15). The RNA-seq data of this study also found that the difference between ASFV-Δ110-9L/505-7R and ASFV-WT was mainly caused by the ability of these two strains to respond to immune responses.

**FIG 4:**
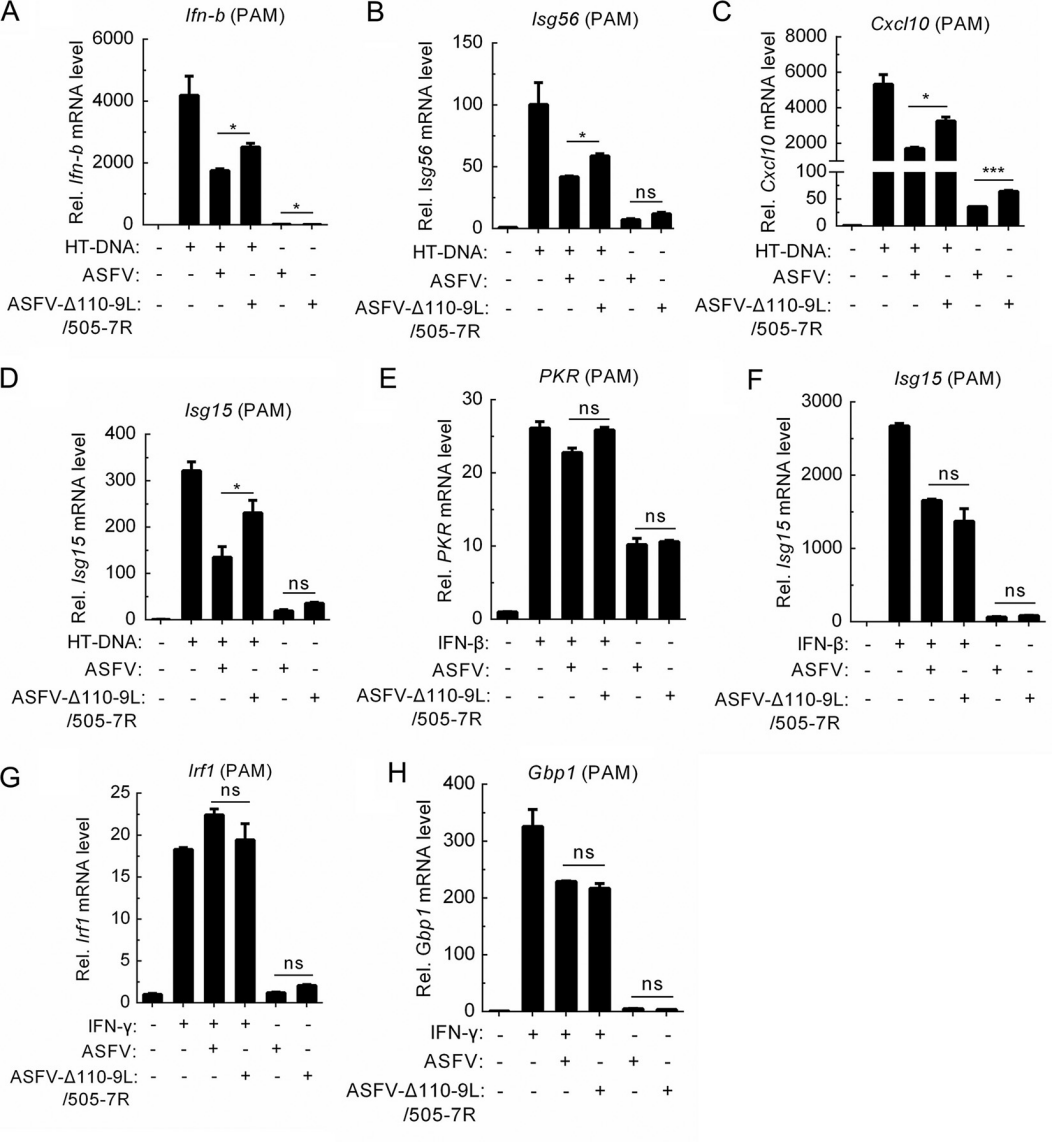
ASFV-WT infection inhibits transcription levels of type I interferon pathway-associated genes compare with ASFV-Δ110-9L/505-7R. (A to D) Effect of ASFV-WT and ASFV-Δ110-9L/505-7R on HT-DNA-induced transcription of *Ifn-b* (A), *Isg56* (B), *Cxcl10* (C), and *Isg15* (D) in PAM cells. PAM cells were uninfected or infected with ASFV-WT and ASFV-Δ110-9L/505-7R at an MOI of 1 for 20 h and then treated with HT-DNA (4 μg/mL) for another 4 h. Cells were collected, and qRT-PCR analysis was performed. (E and F) Effect of ASFV-WT and ASFV-Δ110-9L/505-7R on IFN-β-induced transcription of *PKR* (E) and *Isg15* (F). PAM cells were uninfected or infected with ASFV-WT or ASFV-Δ110-9L/505-7R at an MOI of 1 for 18 h and then treated with IFN-β (1,000 U/mL) for another 6 h. Cells were collected, and qRT-PCR analysis was conducted. (G and H) Effect of ASFV-WT and ASFV-Δ110-9L/505-7R on IFN-γ-induced *Irf1* (G) and *Gbp1* (H) transcription. PAM cells were uninfected or infected with ASFV-WT or ASFV-Δ110-9L/505-7R at an MOI of 1 for 18 h and then treated with IFN-γ (100 ng/mL) for another 6 h. Cells were collected, and qRT-PCR analysis was carried out. qRT-PCR, quantitative real-time PCR; HT-DNA, Herring testis DNA; PAMs, porcine alveolar macrophages. *, *P < *0.05; ***, *P < *0.005; ns, no significance.

To determine whether the attenuated virulence of ASFV-Δ110-9L/505-7R is related to the induction of type I IFN and type II IFN, porcine alveolar macrophage (PAM) cells were first infected with ASFV-WT and ASFV-Δ110-9L/505-7R and then were treated or not treated with Herring testis DNA (HT-DNA, an IFN agonist, activates the cyclic GMP-AMP synthase-STING-dependent IFN signaling pathway) (31). We observed that ASFV-Δ110-9L/505-7R infection increased HT-DNA-triggered transcription levels of *Ifn-b* (Fig. 4A), *Isg56* (Fig. 4B), *Cxcl10* (Fig. 4C), and *Isg15* (Fig. 4D) compared with ASFV-WT. Further, the effect of ASFV-WT and ASFV-Δ110-9L/505-7R on IFN-β- and IFN-γ-stimulated gene expression was also detected, and the results showed that the expression of *Isg15* and *PKR* (as representative IFN-β-induced genes), and *Irf1* as well as *Gbp1* (as representative IFN-γ-induced genes), has no significant difference in ASFV-WT- and ASFV-Δ110-9L/505-7R-infected cells (Fig. 4E to H). These results revealed that ASFV-Δ110-9L/505-7R infection specifically promotes the expression of genes associated with the type I interferon pathway compared with ASFV-WT infection.

### ASFV-Δ110-9L/505-7R infection blocked autophagic degradation of TBK1.

**FIG 5:**
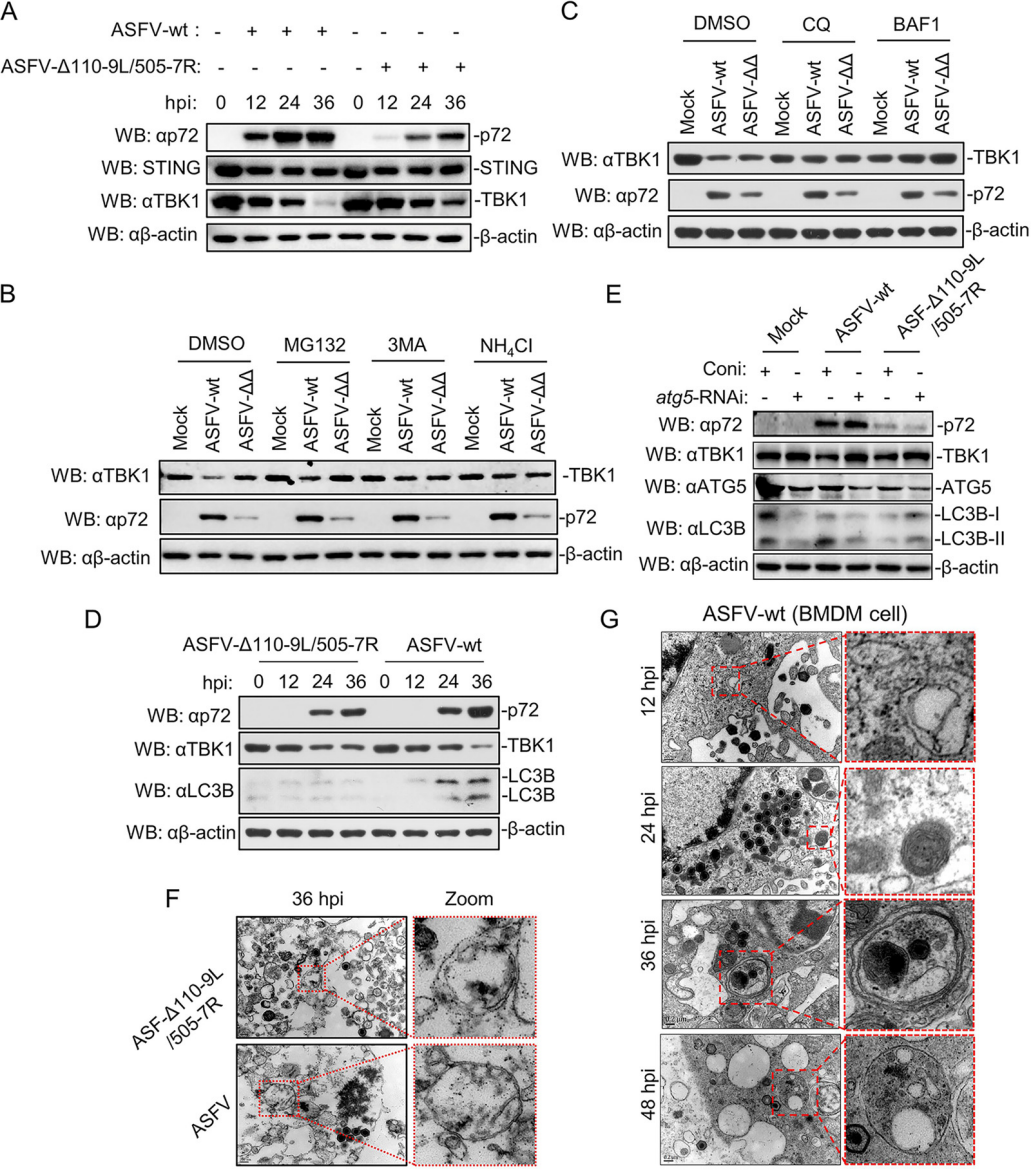
ASFV-Δ110-9L/505-7R weakens TBK1 degradation by the autophagy pathway. (A) Expression of TBK1 and STING in PAM cells infected with ASFV-WT and ASFV-Δ110-9L/505-7R. PAM cells were uninfected or infected with ASFV-WT or ASFV-Δ110-9L/505-7R at an MOI of 1 for the indicated time points, and then cells lysates were analyzed by Western blotting utilizing antibodies STING, TBK1, p72, and β-actin. (B) Effects of inhibitors on ASFV-WT or ASFV-Δ110-9L/505-7R-mediated destabilization of TBK1. PAM cells were uninfected or infected with ASFV-WT or ASFV-ΔΔ (ASFV-Δ110-9L/505-7R) at an MOI of 1 for 20 h. Then, cells were treated with dimethyl sulfoxide (DMSO), proteasome inhibitor MG132 (100 μM), autophagy inhibitor 3-MA (0.5 μg/μL), or lysosome inhibitor NH_4_Cl (25 mM) for another 4 h. Cells were collected, and Western blotting was performed using antibodies TBK1, p72, and β-actin. (C) Effects of inhibitors on ASFV-WT or ASFV-ΔΔ-mediated destabilization of TBK1. PAM cells were uninfected or infected with ASFV-WT or ASFV-ΔΔ at an MOI of 1 for 20 h. Then, cells were treated with DMSO, chloroquine (CQ), and bafilomycin A1 (BAF1) for another 4 h. Cells were collected, and Western blotting was performed using antibodies TBK1, p72m, and β-actin. (D) Effect of ASFV-WT and ASFV-Δ110-9L/505-7R infection on expression of LC3B and TBK1. PAM cells were infected with ASFV-WT or ASFV-Δ110-9L/505-7R (MOI of 1) at 0, 12, 24, and 36 hpi. The expression of p72, p30, TBK1, and LC3B was evaluated by Western blotting utilizing the indicated antibodies. (E) Effect of *ATG5* silence on TBK1 expression. PAM cells were silenced with specific *ATG5* or scrambled siRNA for 48 h and then infected with ASFV-WT and ASFV-Δ110-9L/505-7R for another 24 h. Cellular lysates were detected by Western blotting analysis using antibodies TBK1, *ATG5*, LC3B, p72, and β-actin. (F and G) Electron microscopy of autophagosome formation in ASFV-WT- and ASFV-Δ110-9L/505-7R-infected PAM cells. PAM cells were infected with ASFV-WT or ASFV-Δ110-9L/505-7R (MOI of 1) at 12, 24, and 36 hpi. Cells were fixed, processed for conventional Epon embedding, and observed by transmission electron microscopy.

To address the potential role of ASFV-Δ110-9L/505-7R in regulating the IFN-β signaling pathway, we examined the effects of ASFV-Δ110-9L/505-7R on proteins of cGAS-STING signaling. ASFV-Δ110-9L/505-7R slightly restored TBK1 reduction, but not STING, in PAM cells compared to those in ASFV-WT-infected PAM cells (Fig. 5A). TBK1 degradation in ASFV-WT-infected PAM cells was blocked with autophagy inhibitors 3-MA, chloroquine (CQ), bafilomycin A1 (BAF1), and a lysosome inhibitor NH_4_Cl treatment, but not with a proteasome inhibitor, MG132, treatment (Fig. 5B and C). To further validate that TBK1 degradation was mediated by autophagy pathway, we detected the expression of LC3B (a commonly used autophagy marker) in ASFV-Δ110-9L/505-7R- or ASFV-WT-infected PAM cells. The results showed that TBK1 degradation caused by ASFV infection was positively correlated with autophagy (Fig. 5D), indicating that the TBK1 degradation occurs through the autophagy pathway.

Furthermore, the autophagy pathway was verified by silenced autophagy key protein ATG5 expression, and the results showed that ATG5 silence blocked TBK1 degradation in ASFV-WT- and ASFV-Δ110-9L/505-7R-infected PAM cells (Fig. 5E). Moreover, autophagosome formation was increased in ASFV-WT-infected bone marrow-derived macrophage (BMDM) cells by transmission electron microscopy (TEM) (Fig. 5G). In contrast, autophagosome formation was reduced in ASFV-Δ110-9L/505-7R-infected PAMs compared with that in ASFV-WT-infected BMDM cells (Fig. 5F). Collectively, these results indicated that ASFV-Δ110-9L/505-7R restores TBK1 reduction by inhibiting the autophagy pathway.

### PIK3C2B is an important upregulated molecule during ASFV infection.

**FIG 6:**
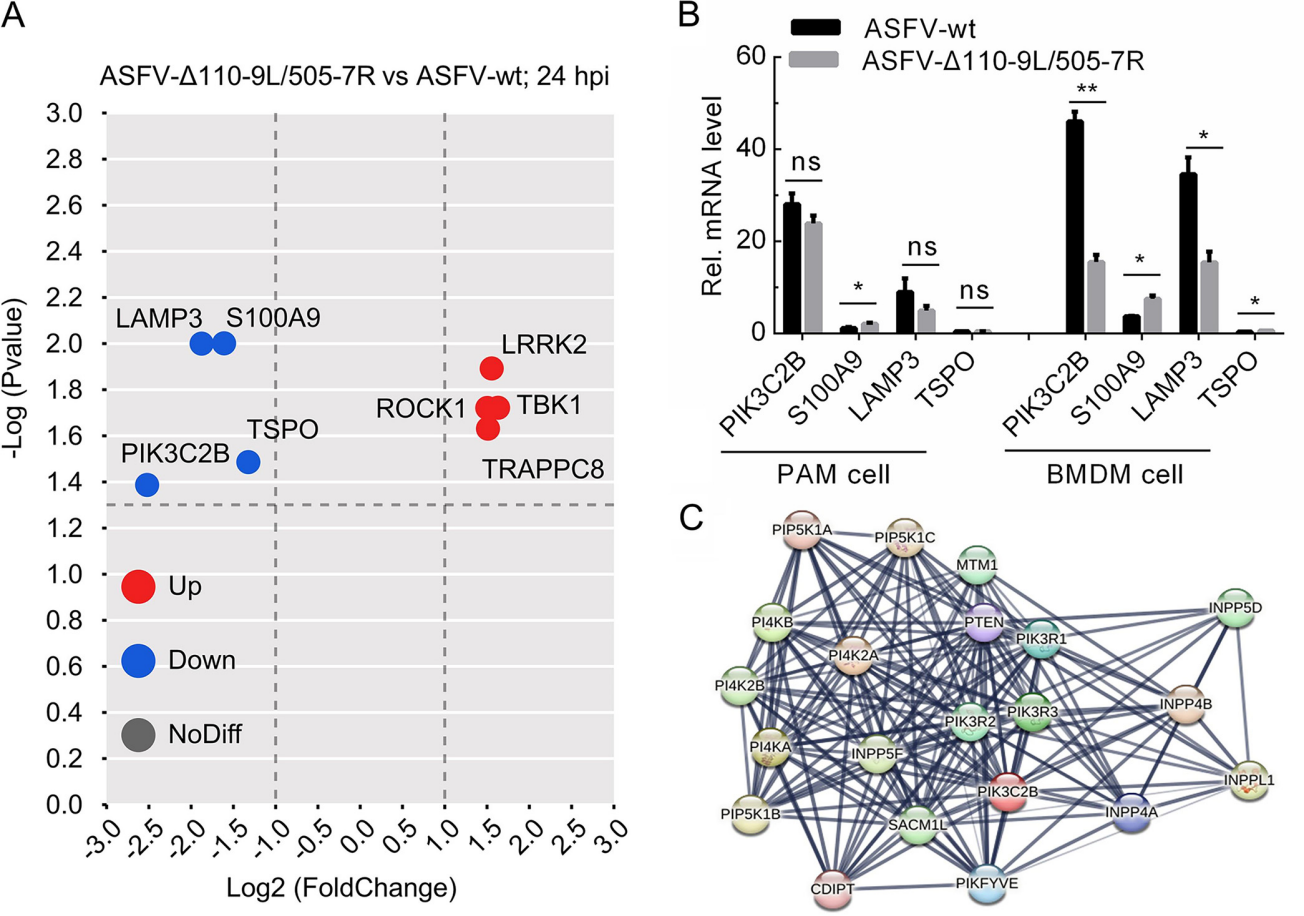
PIK3C2B is one of the important differentially expressed autophagy molecules. (A) Volcano map of DEGs associated with autophagy in ASFV-Δ110-9L/505-7R- and ASFV-WT-infected cells at 24 hpi. The red dots represent the upregulated DEGs, and the blue dots represent the downregulated DEGs. The gray dots indicate no significant difference. (B) qRT-PCR validation for downregulated DEGs in panel A. PAM and BMDM cells were infected with ASFV-WT or ASFV-Δ110-9L/505-7R for 24 h. Cells were collected, and transcription levels of *PIK3C2B*, *S100A9*, *LAMP3*, and *TSPO* were verified by qRT-PCR. (C) The STRING database predicted the PIK3C2B protein and host protein interaction network (https://string-db.org/). PAM, porcine alveolar macrophages; BMDM, bone marrow-derived macrophage; qRT-PCR, quantitative real-time PCR; hpi, hours postinfection. *, *P < *0.05; **, *P < *0.01; ns, no significance.

The above-described results indicated that TBK1 degradation was mediated by the autophagy pathway. Therefore, we next analyzed the differential expression of autophagy-associated genes in ASFV-Δ110-9L/505-7R- and ASFV-WT-infected cells at 24 h. RNA-seq analysis results showed that the expressions of *PIK3C2B*, *LAMP3*, *S100A9*, and *TSPO* in ASFV-Δ110-9L/505-7R-infected cells were downregulated compared that those in ASFV-WT-infected cells. Meanwhile, *LRRK2*, *ROCK1*, *TBK1*, and *TRAPPC8* were upregulated in ASFV-Δ110-9L/505-7R-infected cells compared with those in ASFV-WT-infected cells (Fig. 6A). Of note, PIK3C2B has the largest fold change in these genes (Fig. 6A). The data were further verified by qRT-PCR analysis in infected PAM and BMDM cells. The result showed that the expressions of *PIK3C2B* and *LAMP3* genes in ASFV-WT-infected cells were higher than those in ASFV-Δ110-9L/505-7R-infected cells (Fig. 6B). A previous study has shown that *PIK3C2B* has been confirmed to repress mTOR1 activity and, finally, contribute to autophagy (32). In contrast, *LAMP3* has been reported to inhibit autophagy (33, 34). Therefore, TBK1 degradation should be mediated by *PIK3C2B* upregulation rather than *LAMP3* upregulation. In addition, the protein and protein interaction network of PIK3C2B was predicted via the STRING database (https://string-db.org/), and some potential proteins such as SACM1L (also called SAC1), INPP4B, PIKFYVE, and PTEN, related with autophagy, were included in the interaction network (Fig. 6C) (35–37). Therefore, PIK3C2B is likely to be a critical molecule that mediates the autophagic degradation of TBK1.

### PIK3C2B promoted ASFV-Δ110-9L/505-7R replication.

**FIG 7:**
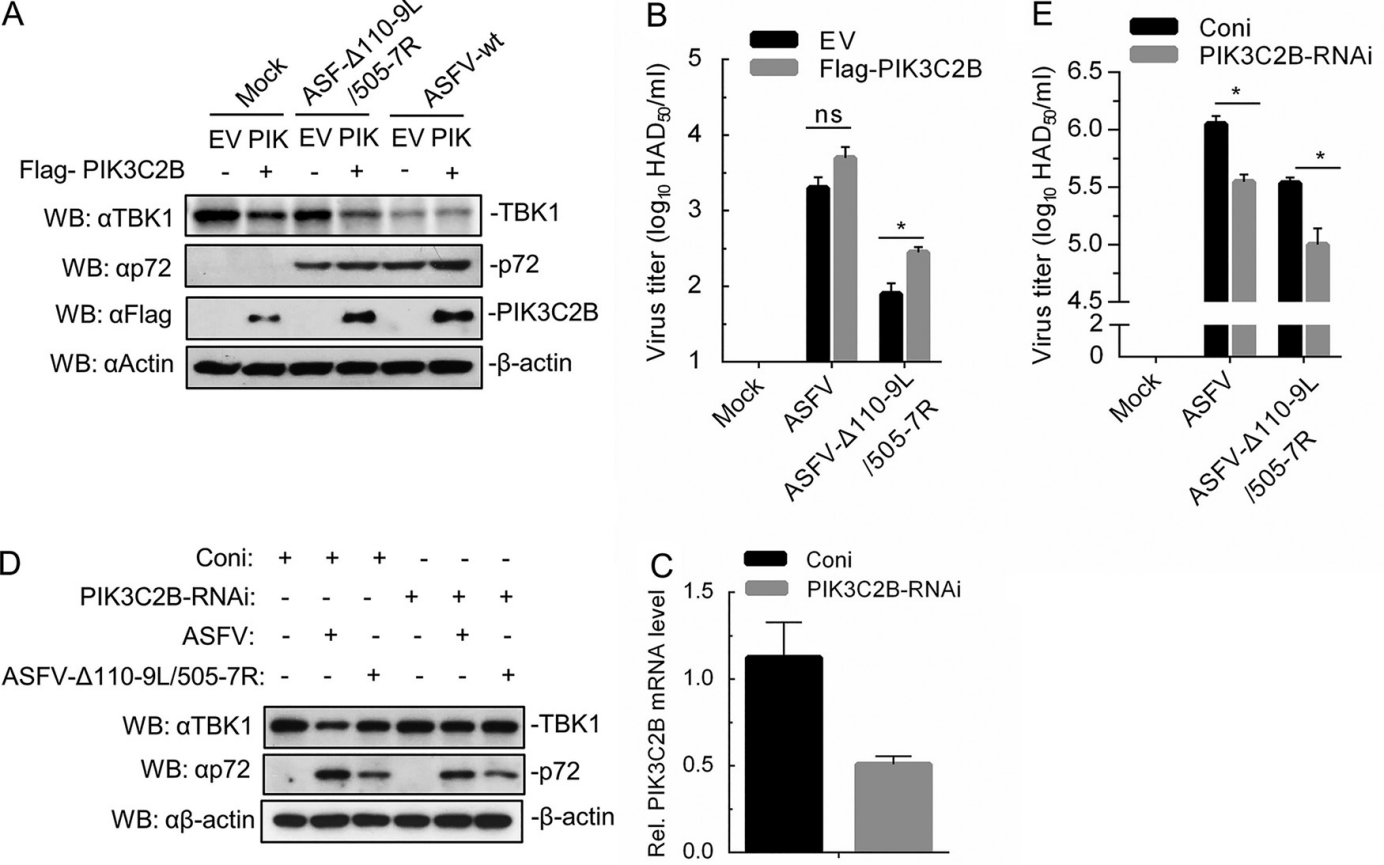
PIK3C2B is a key factor for ASFV-WT infection-induced TBK1 degradation. (A) PIK3C2B overexpression reduces TBK1 expression in ASFV-WT- and ASFV-Δ110-9L/505-7R-infected cells. MA104 cells were transfected with empty vector (EV) or Flag-PIK3C2B expression plasmid for 24 h and then infected with ASFV-WT or ASFV-Δ110-9L/505-7R (MOI of 1) for an additional 24 h. The expression of TBK1, p72, and PIK3C2B was detected by Western blotting with antibodies TBK1, p72, Flag, and β-actin. (B) PIK3C2B overexpression promotes ASFV-Δ110-9L/505-7R replication. MA104 cells were treated by the same method as in panel A. Cells and supernatant were collected for measuring virus titers by HAD_50_ assay. (C and D) PIK3C2B silence restores TBK1 reduction in ASFV-WT- and ASFV-Δ110-9L/505-7R-infected cells. PAM cells were transfected with specific PIK3C2B or scrambled siRNAs for 48 h and then infected with ASFV-WT or ASFV-Δ110-9L/505-7R (MOI of 1) for another 24 h. (C) Cells were collected, and the effect of *PIK3C2B* silence was confirmed by qRT-PCR. (D) The expression of TBK1 and p72 was evaluated by Western blotting with antibodies TBK1, p72, and β-actin. (E) PIK3C2B silence decreases ASFV replication. PAM cells were treated via the same method as in panel C. Virus titer was determined by HAD_50_ assay. qRT-PCR, quantitative real-time PCR; HAD_50_, 50% hemadsorption dose. *, *P < *0.05; ns, no significance.

We next determined whether PIK3C2B expression affected ASFV replication and TBK1 expression. MA104 cells were transfected with PIK3C2B expression plasmid for 24 h and then infected with ASFV-Δ110-9L/505-7R or ASFV-WT for another 24 h. The results showed that PIK3C2B overexpression could decrease the expression of TBK1 and promote the expression of the viral protein p72 (Fig. 7A). The effect of PIK3C2B overexpression on virus titer was verified by 50% hemadsorption dose (HAD_50_) assay, and we found that PIK3C2B promoted ASFV-Δ110-9L/505-7R replication (Fig. 7B). However, no significant difference was observed in ASFV-WT-infected PIK3C2B-overexpressed cells (Fig. 7B).

To further investigate the roles of endogenous PIK3C2B in ASFV replication, PIK3C2B expression was silenced by specific small interfering RNA (siRNA) (Fig. 7C). Western blotting results showed that endogenous PIK3C2B reduction restored TBK1 degradation in ASFV-WT- and ASFV-Δ110-9L/505-7R-infected PAM cells and inhibited the expression of p72 (Fig. 7D). Moreover, knockdown of PIK3C2B reduced virus titer in ASFV-WT- and ASFV-Δ110-9L/505-7R-infected PAM cells (Fig. 7E). These observations suggest that PIK3C2B can increase ASFV-Δ110-9L/505-7R replication and regulate TBK1 degradation.

## DISCUSSION

ASFV is the sole member of the family *Asfarviridae* and belongs to nucleocytoplasmic large DNA viruses (NCLDVs) (38, 39). Interestingly, inactivated ASFV vaccine candidates did not induce protective immunity in pigs (40). Recently, significant advances have been achieved in several ASF vaccines, including inactivated vaccines, live attenuated vaccines (LAVs), subunit vaccines, DNA vaccines, and viral vector vaccines (1–3). Of these, LAVs (ASFV-GZ-ΔI177L, HLJ/18-7GD, SY18ΔI226R, and ASFV-G-ΔA137R, etc.) (25, 29, 41–43) are the only vaccines that induce 100% immune protection against parental ASFV challenge. However, LAVs carry the risk of virus shedding and field virus recombination (29), which is a huge biosecurity challenge and threat for long-term ASF prevention and control.

Previous studies have shown that gene-deleted LAVs of ASFV were still the most effective vaccine candidates to prevent ASF (44, 45). Therefore, analyzing and clarifying the differences between LAVs and the parental virus strain are significant for vaccine development (46). This study used RNA-seq analysis to evaluate the transcription difference of genes in PAM cells during ASFV-Δ110-9L/505-7R or ASFV-WT infection. The RNA-seq results showed that DEGs were mainly enriched in the innate immune pathway, and the TBK1 protein, an important adaptor molecule of the type I interferon pathway, was further identified as an obvious DEG involved in innate immune signaling pathways. Finally, PIK3C2B, a positive regulator of autophagy (32), is identified as one of the key molecules mediating differential expression of TBK1 during ASFV-Δ110-9L/505-7R and ASFV-WT infection. This study provides new ideas for the study of LAV pathogenesis and has important significance for developing ASF vaccines.

However, several issues need to be raised and discussed in this study as follows. (i) Previous studies have shown that the deletion of genes from different strains or isolates does not always have the same outcomes (19, 47), and several different ASFV strains (including genotype I ASFV) are circulating in China (48–52). This study only focused on the type II ASFV strain (ASFV CN/GS/2018 isolate). Whether this study's results are consistent with the type I strain or other type II ASFV strains is necessary for further studies. (ii) Thus far, only LAVs have shown solid protection (some LAVs provided protection rates as high as 100%) against the lethal ASFV-WT challenge (28). An effective ASF vaccine must be accompanied by an effective protective immune mechanism and require cross-involvement of both innate and adaptive immune cross talk (53). A previous study has found that MGF505-7R is not only involved in regulating the innate immune response (19) but also can be recognized by specific T cells (54). In future studies, we should pay more attention to illustrating the relationship and mechanism between innate and adaptive immune response, which plays an important role in understanding the immune protection mechanisms of ASFV-Δ110-9L/505-7R. (iii) Previous studies have shown cell apoptosis is necessary for ASFV pathogenesis (55) and inflammatory response is regulated during ASFV infection (56, 57). This study mainly illustrated the interferon pathway (Fig. 4) in the immune response. Although the innate response is the most significant signaling pathway for GO enrichment, there is also enrichment in other pathways. Therefore, the influence of other factors on virus virulence is our future research direction.

In summary, this study reveals that the attenuated virulence of ASFV-Δ110-9L/505-7R is probably associated with restoring TBK1 autophagic degradation. TBK1 autophagic degradation is further identified to be regulated by a positive autophagy regulation molecule, PIK3C2B. Our findings show the involvement of the PIK3C2B-TBK1 axis in innate immune responses during ASFV infection, providing important clues for exploring and understanding the mechanisms of mutant ASFV strain infection. Notably, the identification of MGF110-9L and MGF505-7R as important inhibitors of innate immune responses and critical determinants of ASFV virulence points provides a new host-virus interaction mechanism of ASFV-Δ110-9L/505-7R and theoretical guidance for the development of ASF vaccines.

## MATERIALS AND METHODS

### Ethics statements.

All experiments involved with ASFV-WT and ASFV-Δ110-9L/505-7R were conducted in the biosafety level 3 (BSL-3) facilities in the Lanzhou Veterinary Research Institute (LVRI) of the Chinese Academy of Agricultural Sciences, approved by the Ministry of Agriculture and Rural Affairs.

### Cell cultures and viruses.

Porcine alveolar macrophages (PAMs) were prepared from the lung lavage fluid of 30-day-old pigs as previously described (58) and then cultivated in RPMI 1640 medium containing 10% fetal bovine serum (Biological Industries [BI]) and 1% penicillin-streptomycin at a humidified 37°C and 5% CO_2_ atmosphere. The ASFV-WT isolate and ASFV-Δ110-9L/505-7R, preserved at the LVRI, CAAS, have been described before (15).

### Antibodies and reagents.

Rabbit polyclonal antibodies against p72 and p30 were prepared in our laboratory (13). Anti-STING (catalog no. MABF270), anti-β-actin (catalog no. A5441), and anti-Flag (catalog no. F2555) monoclonal antibodies (MAbs) were obtained from Sigma-Aldrich. Anti-TBK1 (catalog no. 38066S), anti-LC3B (catalog no. 3868S), and anti-ATG5 (catalog no. 9980S) MAbs were purchased from Cell Signaling Technology. Dimethyl sulfoxide (DMSO), 3-MA (autophagosome formation inhibitor), MG132 (proteasome inhibitor), and NH_4_Cl (lysosome inhibitor) were purchased from Sigma-Aldrich.

### Quantitative real-time PCR and RNA interference experiments.

Total cellular RNA was isolated with TRIzol (Invitrogen Life Technologies) reagent from the treated cells, and then RNA was transcribed to cDNA using a reverse transcription kit (TaKaRa; code no. RR047A). Transcription levels of genes were assessed by qRT-PCR analysis using the SYBR green premix (Bio-Rad) following the manufacturer's instructions. Gene expression levels were normalized to GAPDH (glyceraldehyde-3-phosphate dehydrogenase), and relative mRNA expression levels were evaluated and calculated utilizing the threshold cycle (2^−ΔΔ^*^CT^*) method. All primers used in the qRT-PCR assay are listed in Table 3. Small interfering RNAs (siRNAs) and siRNA-control were designed and synthesized by Sangon Biotech (Shanghai, China). PAM cells were transfected with ATG5 siRNA for 48 h and then infected with ASFV-WT and ASFV-Δ110-9L/505-7R for the indicated time points at a multiplicity of infection (MOI) of 1. Expression of proteins and knockdown efficiency were detected by Western blotting and qRT-PCR analyses, respectively.

**TABLE 3:** Information on primers used in this study

Primer name	Forward sequence (5′–3′)	Reverse sequence (5′–3′)
sus-*INF-β*	CACTGGCTGGAATGAAACCG	AATGGTCATGTCTCCCCTGG
sus-*ISG56*	TCAGAGGTGAGAAGGCTGGT	GCTTCCTGCAAGTGTCCTTC
sus-*ISG15*	CCTGTTGATGGTGCAAAGCT	TGCACATAGGCTTGAGGTCA
sus-*CXCL10*	CTGTTCGCTGTACCTGCATC	GCTTCTCTCTGTGTTCGAGG
sus-*IL-1β*	AAGATGACACGCCCACCCTG	TACCAGTTGGGGTACAGGGCA
sus-*GBP1*	GAAGGGTGACAACCAGAACGAC	AGGTTCCGACTTTGCCCTGATT
sus-*TBK1*	GCTGGCTGATACATGGACCC	GCTAGTCTACGTTCTGCTTTGTC
sus-*TNF-α*	GCCCAAGGACTCAGATCATC	GGCATTGGCATACCCACTCT
sus-*IRF1*	TCCAGCCGAGATGCTAAGTG	GTCCAAGTCCTGCCCGATGT
sus-*PKR*	ATTGCGAGAAGGTAGAGCGT	TTCCATTTGGATGAAAAGGCACC
sus-*TSPO*	CTCACGCAATGTCCTCGGAA	CATACCCCATGGCCGAGTAG
sus-*LAMP3*	TGGAAATGTGGACGAGTGTTCA	AGACAATCAAACCCACAGCAC
sus-*S100A9*	GGCTTGGTGATAGGAGGTGTC	GCACTCTGTCTGAGCAATGGA
sus-*pik3c2b*	TGCGCTTTACTCCAAATGGC	CCAGTTACGGAATGAGTCAGCA
sus-*GAPDH*	ACATGGCCTCCAAGGAGTAAGA	GATCGAGTTGGGGCTGTGACT
*MGF505-7R*	GACTGGCATGTTCTCCTCCC	ACAGCATGTTGGAGAAGGCA
*MGF110-9L*	CGTGTCGACAATTCCAGCAG	TCCTTTTGGTACTGGCGGTC

### Western blotting.

The transfected or virus-infected cells were washed twice with cold phosphate-buffered saline (PBS), lysed in lysis buffer (pH 7.5, 50 mM Tris-HCl, 150 mM NaCl, 0.5% Triton X-100, 10% glycerol, 2% SDS, β-mercaptoethanol, bromophenol blue, and 1 mM EDTA), and heated at 90°C for 10 min. Then proteins were separated with SDS-PAGE and transferred to the nitrocellulose (NC) membrane (Pall). Transferred membranes were incubated overnight at 4°C with the primary antibodies and for 1 h at room temperature (RT) with the corresponding horseradish peroxidase-conjugated secondary antibodies. Proteins were detected with Pierce ECL Western blotting substrate (Thermo Fisher Scientific).

### RNA sequencing analysis.

PAM cells were mock infected or infected with ASFV-WT and ASFV-Δ110-9L/505-7R strains at indicated time points (0, 5, 12, and 24 hpi). Cells were harvested for RNA extraction using TRIzol reagent (Invitrogen, CA, USA). RNA integrity was assessed using the Agilent 2100 Bioanalyzer (Agilent Technologies, Santa Clara, CA, USA). Then, the libraries were constructed using VAHTS universal V6 RNA-seq library prep kit according to the manufacturer's instructions. The transcriptome sequencing and analysis were conducted by OE Biotech Co., Ltd. (Shanghai, China). Finally, differential expression genes (DEGs) in the PAM cells infected with ASFV-WT and ASFV-Δ110-9L/505-7R were identified utilizing the edgeR R package (3.18.1). The *P* values were adjusted using the Benjamini-Hochberg method. A corrected *P* value of <0.05 and absolute fold change (|log_2_FC|)of >2 were set as the thresholds for significantly differential expression.

### HAD_50_ assay.

HAD_50_ assay was used to quantify the median tissue culture infectivity of ASFV-WT- and ASFV-Δ110-9L/505-7R-infected samples as previously described, with a minor modification (59). Briefly, PAM cells were seeded in 96-well plates for 2 h, and then 10-fold serially diluted samples were added into each well in triplicate. Subsequently, cells were incubated with 2% porcine red blood cells diluted in PBS, and the hemadsorption (HAD) phenomenon was observed for 5 to 7 days. Finally, HAD_50_ was calculated utilizing the Reed and Muench method.

### Transmission electron microscopy.

For transmission electron microscopy (TEM) analysis of autophagosome, ASFV-WT- and ASFV-Δ110-9L/505-7R-infected PAM cells were fixed with 2.5% glutaraldehyde (Merck, Germany) in 0.1 M phosphate buffer for 1 h at RT. Cells were then postfixed with 2% osmium tetroxide and embedded in epoxy according to standard procedures. After polymerization, about 80-nm-thick sections were obtained and stained with uranyl acetate and lead citrate as previously described (60). Samples were observed under a transmission electron microscope (HT7700; Hitachi, Japan) operated at 80 kV.

### Statistical analysis.

All experiments were done at least in duplicate for a total of ≥3 biological replicates. Data are displayed as means ± standard deviation (SD). Statistical significance was determined using the unpaired two-tailed Student's *t* test. Comparisons showing a *P* value of <0.05 were considered statistically significant. Asterisks indicate the statistical significance between connected two bars; *, *P < *0.05; **, *P < *0.01; ***, *P < *0.005; and ****, *P < *0.001.

### Data availability.

The RNA sequencing results were deposited in the Sequence Read Archive database (BioProject accession no. PRJNA952836).
